# Fluorescent humanized anti-CEA antibody specifically labels metastatic pancreatic cancer in a patient-derived orthotopic xenograft (PDOX) mouse model

**DOI:** 10.18632/oncotarget.26484

**Published:** 2018-12-18

**Authors:** Thinzar M. Lwin, Kentaro Miyake, Takashi Murakami, Jonathan C. DeLong, Siamak Amirfakhri, Filemoni Filemoni, Sang Nam Yoon, Paul J. Yazaki, John E. Shivley, Brian Datnow, Bryan M. Clary, Robert M. Hoffman, Michael Bouvet

**Affiliations:** ^1^ Department of Surgery, University of California San Diego, San Diego, CA, USA; ^2^ AntiCancer, Inc., San Diego, CA, USA; ^3^ Department of Gastroenterological Surgery, Yokohama City University, Graduate School of Medicine, Yokohama, Japan; ^4^ VA San Diego Healthcare System, San Diego, CA, USA; ^5^ Department of Molecular Imaging and Therapy, City of Hope, Duarte, CA, USA; ^6^ Department of Pathology, University of California San Diego, San Diego, CA, USA

**Keywords:** fluorescence-guided surgery, LICOR IRDye800CW, anti-CEA antibody, patient derived orthotopic xenograft model, pancreatic cancer

## Abstract

Pancreatic cancer is a highly lethal disease in part due to incomplete tumor resection. Targeting by tumor-specific antibodies conjugated with a fluorescent label can result in selective labeling of cancer *in vivo* for surgical navigation. In the present study, we describe a patient-derived orthotopic xenograft model of pancreatic cancer that recapitulated the disease on a gross and microscopic level, along with physiologic clinical manifestations. We additionally show that the use of an anti-CEA antibody conjugated to the near-infrared (NIR) fluorescent dye, IRDye800CW, can selectively highlight the pancreatic cancer and its metastases in this model with a tumor-to-background ratio of 3.5 (SEM 0.9). The present results demonstrate the clinical potential of this labeling technique for fluorescence-guided surgery of pancreatic cancer.

## INTRODUCTION

Pancreatic cancer is a recalcitrant malignancy. Complete surgical resection with negative margins remains the only curative option. Despite best attempts at pre-operative localization through cross sectional imaging and intra-operative use of anatomic boundaries, surgeons still rely on visual inspection and palpation to determine the location of the lesion and set transection margins [1]. However, most pancreatic resections are incomplete as these visual and tactile cues may miss the tumor margin as well as small metastases, especially sub-centimeter lesions not detectable by pre-operative imaging modalities and this has an impact on patient outcomes [2–5].

Fluorescence guided surgery (FGS) using near-infrared fluorophores (NIR) conjugated to tumor-specific antibodies can assist in visualization of the pancreatic cancer and any intra-abdominal metastases that would preclude the patient from an invasive and ineffective surgical procedure [6]. NIR fluorophores have increased tissue depth penetration while limiting light scattering, absorbance, and auto-fluorescence [7]. Current FDA approved NIR fluorophores include indocyanine green and methylene blue. However, these dyes are non-specific. Conjugation of NIR-fluorophores to antibodies allows selective labeling of tumors and metastases [8].

Studies have been performed with a variety of fluorescently labeled antibodies using cell lines over-expressing the target antigen as a proof of principle [9–12]. However, the tumor microenvironment is a heterogenous population of cells and the ability of fluorescent antibodies to deliver an intense fluorescence signal may be affected. The patient derived orthotopic xenograft (PDOX) models are mouse tumor models that use fresh tumor specimen obtained from patients at the time of surgery, implanted into the corresponding organ of nude mice. They retain the heterogeneity of the patient tumor microenvironment and better represent the physiology and natural biology of the disease, making them a clinically relevant model for optical fluorescence tumor imaging [13–15].

In the present study, we use a human pancreatic cancer PDOX mouse model that recapitulates the clinical behavior of metastatic pancreatic cancer [16]. We show that a fluorescent humanized anti-CEA antibody specifically labels the primary neoplasm as well as sub-millimeter satellite lesions that would otherwise be missed by bright-light imaging and the naked eye.

## RESULTS

### Ascites & jaundice in pancreatic cancer PDOX mice

**Figure 1:**
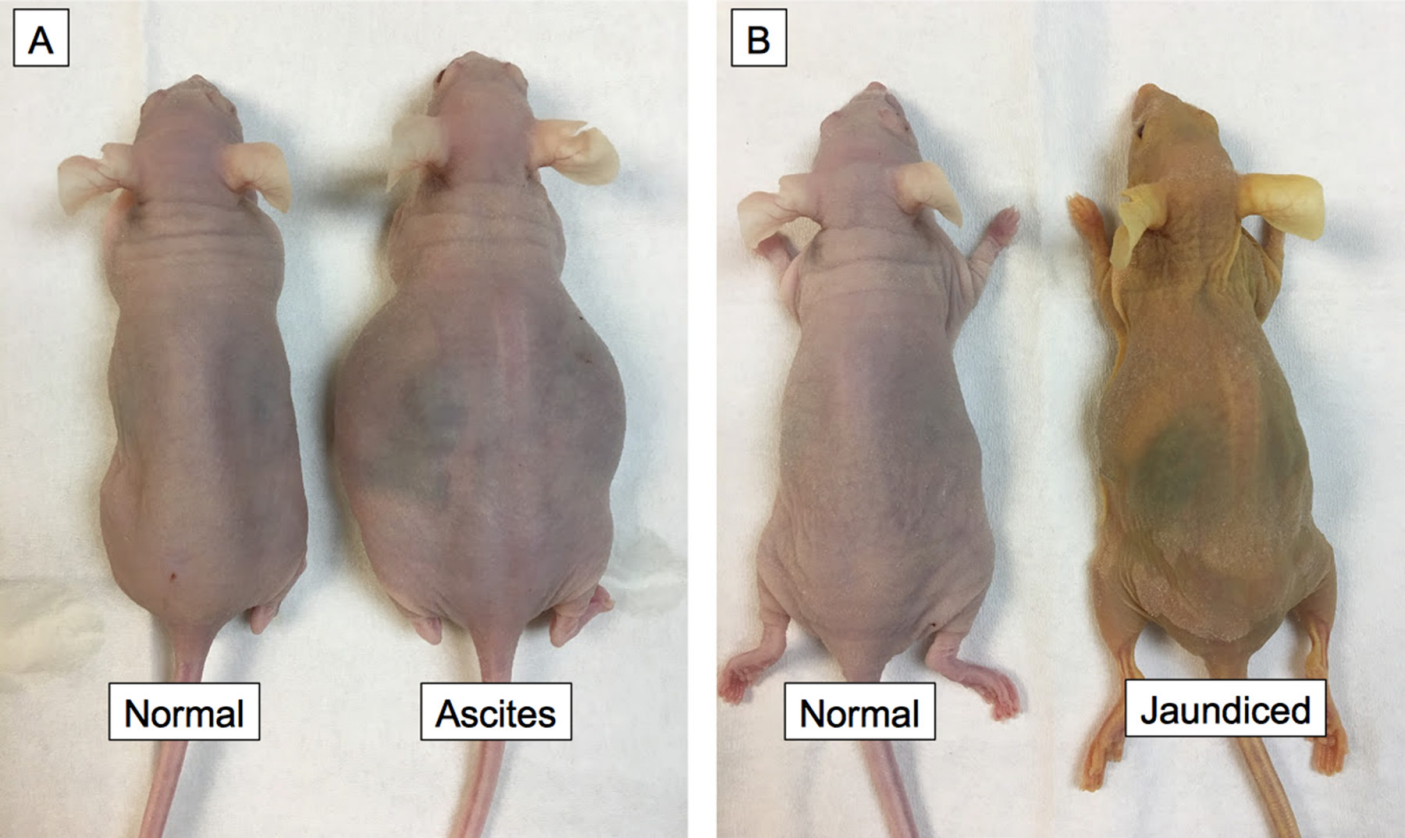
Pancreatic cancer PDOX model leads to ascites and jaundice in mice 2/10 mice developed ascites as evidenced by abdominal distension and weight gain 10–12 weeks after SOI (**A**). One mouse also developed jaundice and cachexia as evidenced by skin color changes and decreased weight14 weeks after SOI (**B**).

The pancreatic cancer PDOX mice in the establishment cohort were observed long term for evidence of metastases (*n* = 10). Palpable primary tumors appeared in all mice by 6 weeks. Two developed ascites as evidenced by abdominal distension and weight gain 10–12 weeks after surgical orthotopic implantation (SOI) (Figure 1A). One mouse also developed jaundice and cachexia as evidenced by skin color changes and weight loss 14 weeks after SOI (Figure 1B). All mice that did not develop ascites showed evidence of cachexia and weight loss greater than 20% of initial weight after 14–16 weeks and had to be sacrificed. The ten mice evaluated long term had diverse metastatic tumors. Supplementary Table 1 outlines the number and location of these lesions.

### Characterization of pancreatic cancer PDOX

**Figure 2:**
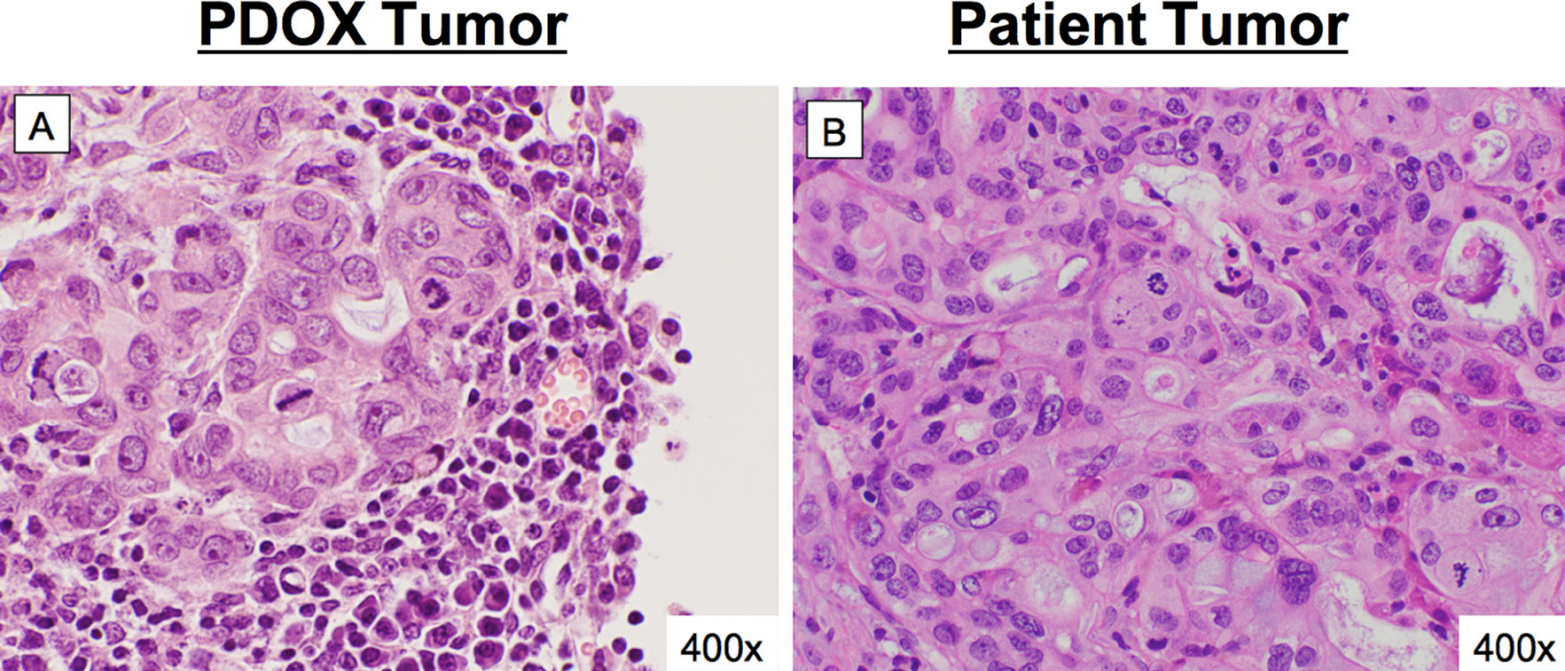
Concordance between patient histology and pancreatic cancer PDOX Microscopic comparisons between the PDOX tumor (**A**) and slides from the patient tumor (**B**) showed consistency in primary and secondary histo-architecture between the two specimens. Both were high grade pancreatic adenocarcinoma.

Histology of harvested pancreatic cancer PDOX orthotopic tumor was that of a high-grade pancreatic adenocarcinoma as seen on henatoxylin and eosin-stained sections (Figure 2A). Microscopic comparisons between the PDOX tumor and slides from the patient tumor showed similarities in primary and secondary histo-architecture (Figure 2A and 2B).

Higher magnification images of the PDOX tumor showed areas invading into normal pancreatic acini (Supplementary Figure 1A and 1B). Within the tumor, there are intermittent areas of glandular formation and mucin production, with diffuse sheets of cancer cells prominent (Supplementary Figure 1B). There were pancreatic ducts with crowded vesicular nuclei and an area of invasion of the myoepithelial layer (Supplementary Figure 1C, black arrowhead). Multiple mitotic figures are present (Supplementary Figure 1D, red arrows).

**Figure 3:**
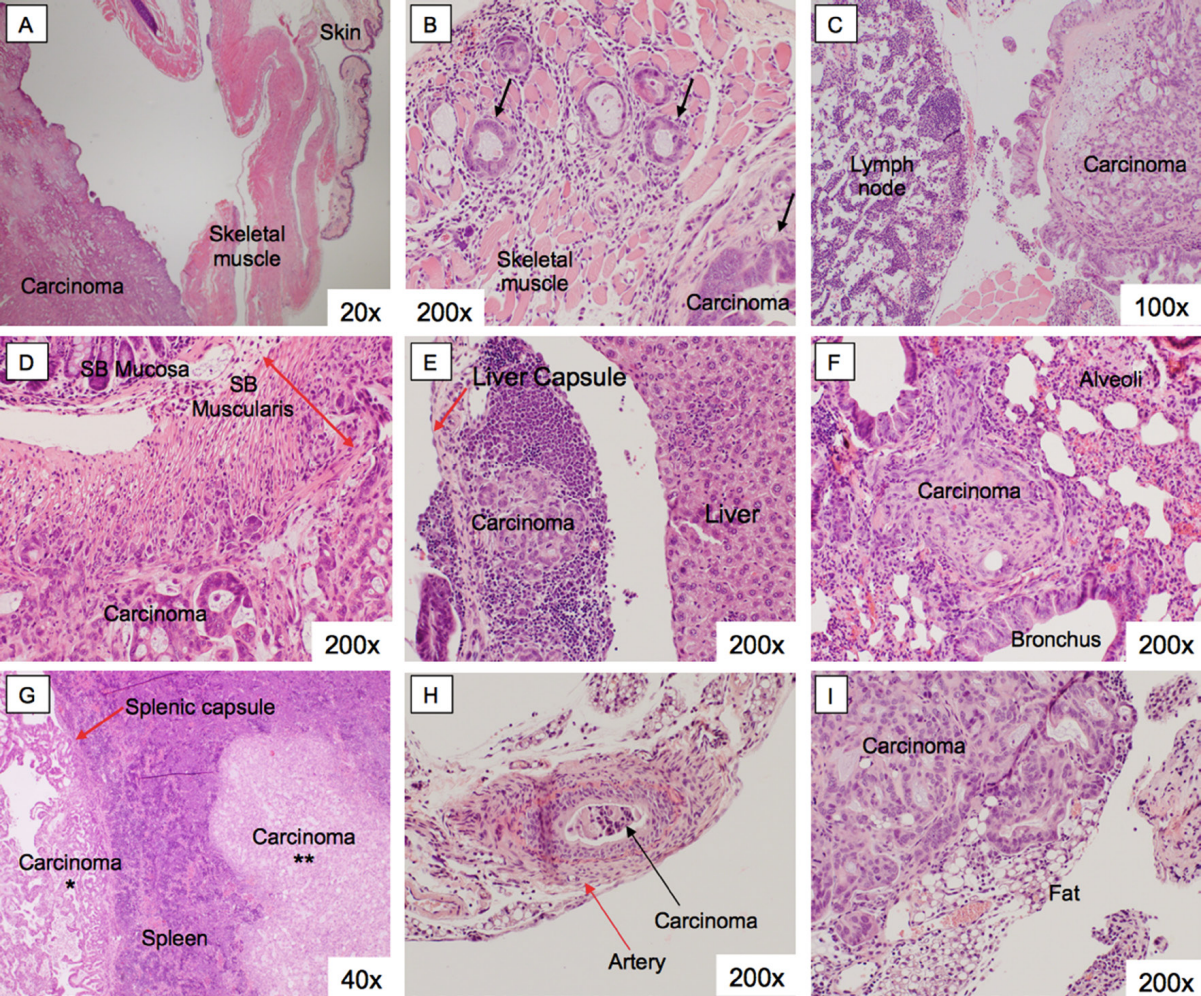
Histology of pancreatic cancer PDOX metastases Organs from PDOX mice with gross intra-abdominal metastases were harvested. There were metastatic deposits seen in the abdominal wall (**A, B**), within the peritoneum (**C**), small bowel (**D**), liver (**E**), lung (**F**), spleen (**G**), artery (**H**), and the retroperitoneum (**I**). These lesions retained the histoarchitecture of the primary tumor.

Upon necropsy, six mice had grossly visible intra-abdominal metastases. Organs were harvested and further examined under light microscopy, representative images are shown. Two mice had microscopically positive metastases in the abdominal wall and spleen (Supplementary Table 1). One mouse had a microscopically positive metastasis in the lung. An abdominal wall deposit is shown in Figure 3A and 3B. The overlying skin and subcutaneous tissue are unremarkable (Figure 3A). There were multiple areas of direct tumor invasion into the skeletal muscle on higher magnification (Figure 3B, black arrows). A peritoneal implant and a lymph node are shown in Figure 3C. This peritoneal implant is a discrete nodule, detached from surrounding abdominal structures. The lymph node does not have any evidence of gross or micro-metastases. Small bowel tumor implants are shown in Figure 3D. Within the small bowel wall, the mucosa is intact, but the tumor invaded into both layers of inner circular and outer longitudinal layers of the smooth muscle. The small bowel muscularis layer is demarcated by the red double headed arrow while the invasive front of the tumor is indicated by the blue dashed line. There were liver deposits within the hepatic capsule (Figure 3E, arrow), but not yet directly invading into the adjacent normal appearing hepatic parenchyma. Although there were no grossly visible deposits on the lung, serial sections showed micro-metastases within the pulmonary parenchyma, between two bronchi (Figure 3F). The spleen showed evidence of both surface deposits outside the splenic capsule (Figure 3G, single asterisk), as well as direct splenic parenchymal invasion (Figure 3G, double asterisk). There was a thick-walled muscular artery with tumor cells within the lumen, evidence of vascular invasion (Figure 3H). A retroperitoneal implant is shown in Figure 3I with invasion of the adjacent adipose tissue.

**Figure 4:**
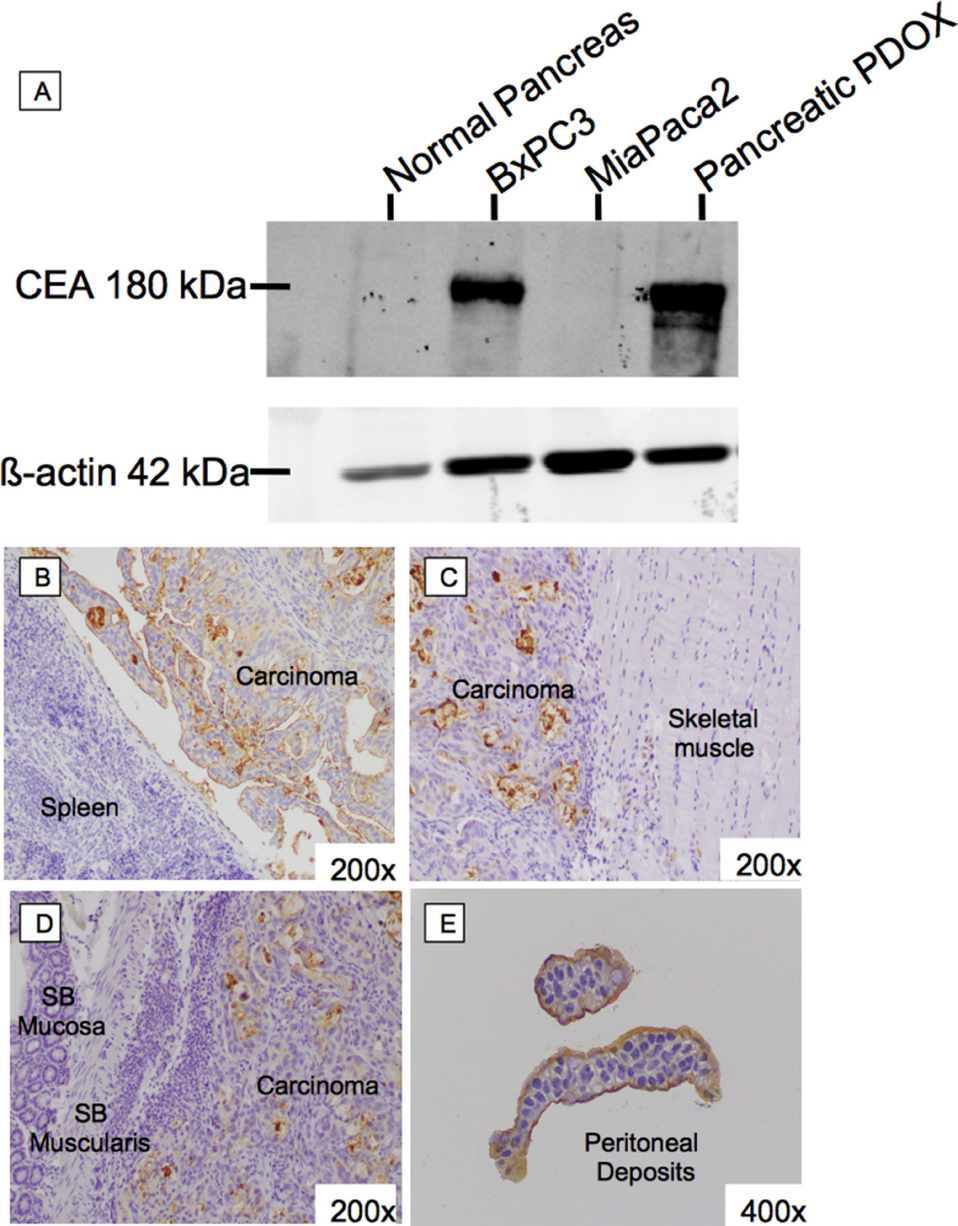
CEA expression of pancreatic cancer PDOX on western blot and immunohistochemistry Western blot of tumor lysates was performed using hM5A as the primary antibody (**A**). There was no CEA staining with tissue lysate from normal pancreatic tissue. Tumor lysates from the CEA positive and negative pancreatic cancer cell line derived tumors show positive and negative staining as expected. Lysates of pancreatic cancer PDOX tissue show strong CEA staining. Immunohistochemistry of Pancreatic Cancer PDOX. Immunohistochemical staining for CEA using hM5A showed strong apical and some cytoplasmic staining at the primary tumor (**B**) as well as abdominal wall (**C**), small bowel (**D**), and peritoneal implants (**E**).

Western blot of tumor lysates was performed using the humanized anti-CEA hT84.66-M5A (hM5A) monoclonal antibody as the primary antibody (Figure 4A). There was no CEA staining observed with tissue lysate from normal pancreatic tissue. Lysates of the pancreatic cancer PDOX tissue shows strong CEA staining.

Immunohistochemical staining for CEA using hM5A shows strong apical and some cytoplasmic staining at the primary tumor (Figure 4B) as well as abdominal wall (Figure 4C), small bowel (Figure 4D), and peritoneal metastases (Figure 4E).

### *In-vivo* fluorescence imaging of pancreatic cancer PDOX

**Figure 5:**
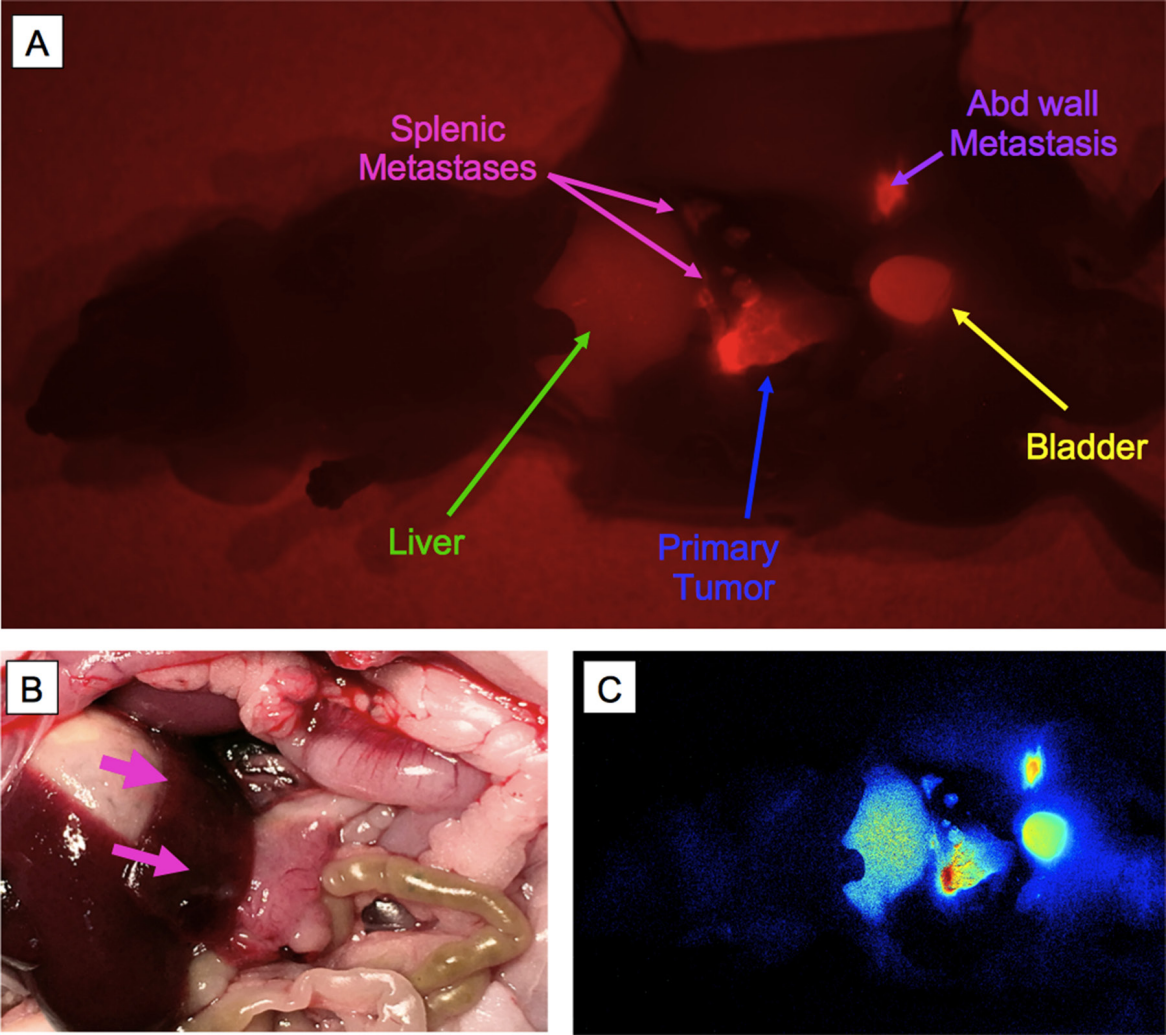
Selective tumor labeling of pancreatic cancer PDOX by hM5A-LICOR800 Optical *in-vivo* imaging using fluorescent M5A-IR800 shows that the antibody-fluorophore conjugate was able to clearly and specifically label the primary pancreatic tumor (**A**, outlined in blue) as well as metastases over the spleen (A, outlined in pink) and abdominal wall (A, purple arrows). There was signal present in the liver and the bladder. A bright light image of the abdomen shows the primary tumor (**B**, outlined in blue dashed lines) and splenic metastases (B, outlined in pink and pink arrows). A fluorescence intensity heat map is displayed in (**C**), showing the most intense fluorescence signal over the primary tumor and abdominal wall metastases.

Fluorescence *in-vivo* imaging using the hM5A conjugated to an IR800 fluorophore shows that the antibody-fluorophore conjugate was able to clearly and specifically label the primary pancreatic PDOX tumor (Figure 5A, outlined in blue dashed line) as well as metastases over the spleen (Figure 5A, outlined in pink fine dashed line) and abdominal wall (Figure 5A, purple arrows). There was signal present in the liver and the bladder. A bright light image of the abdomen shows the primary PDOX tumor (Figure 5B, outlined in blue dashed lines) and splenic metastases (Figure 5B, outlined in pink fine dashed line and arrows). A fluorescence intensity heat map is displayed in Figure 5C, showing the most intense fluorescence signal over the primary tumor and abdominal wall metastases.

### Fluorescence intensity

**Figure 6:**
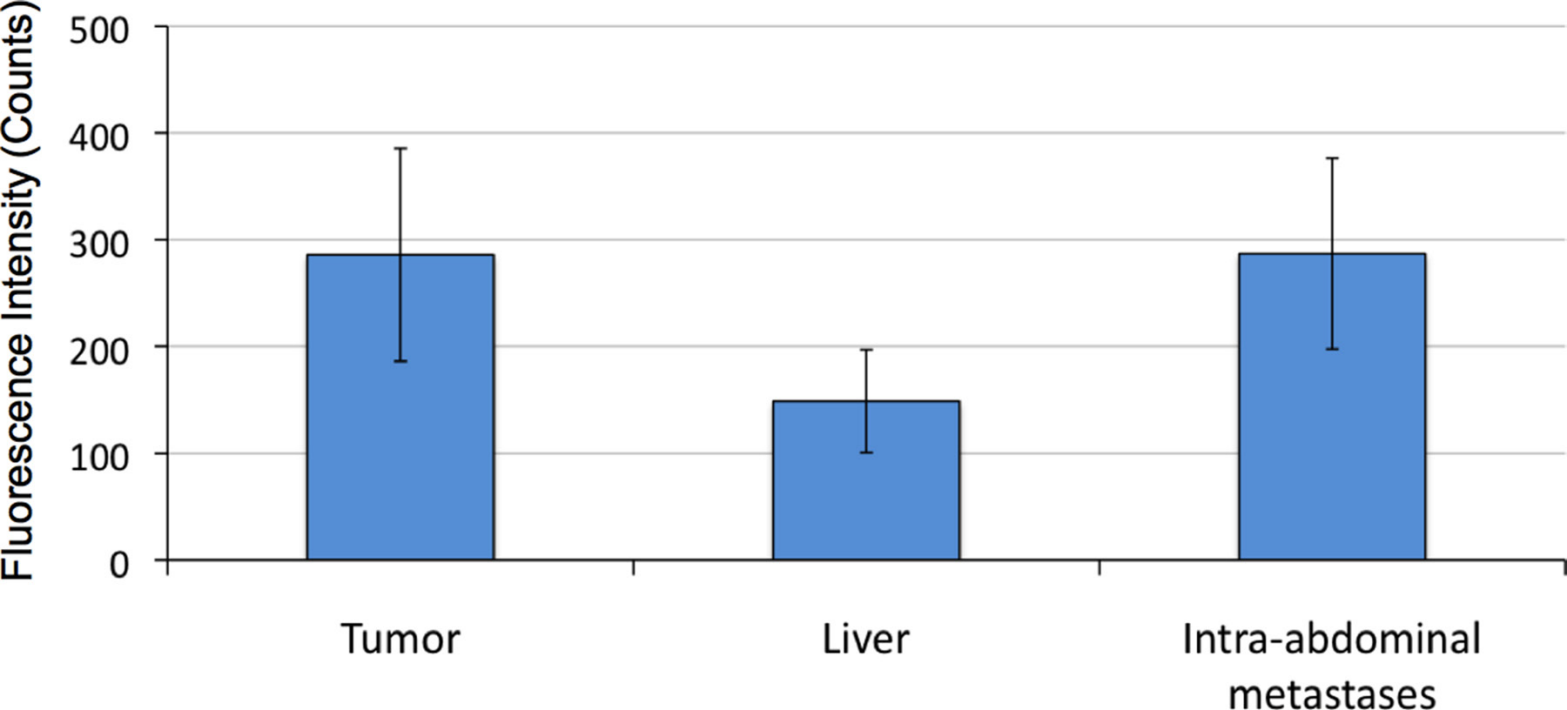
Fluorescence intensity of pancreatic cancer PDOX labeling by hM5A-IR800 Quantification of peak fluorescence signal at 48 hours was measured and adjusted for the background signal at the skin. The average fluorescence intensity after 48 hours was 286 counts from the tumor, 287 counts from the abdominal wall metastases, and 149 counts from the liver (+/– SEM as error bars).

Quantification of peak fluorescence signal at 48 hours was measured and adjusted for the background signal of the skin. The average fluorescence intensity after 48 hours was 286 counts from the tumor, 287 counts from the abdominal wall metastases, and 149 counts from the liver. The fluorescence signal over the splenic metastases was less than that of the tumor and the abdominal wall, but the lesions were too small to quantify reliably. The average tumor to background ratio was 3.5 (SEM 0.9). The results are summarized in Figure 6 ± SEM as error bars.

## DISCUSSION

In the present study, we described a PDOX model of metastatic pancreatic cancer established from a patient surgical specimen and used this PDOX model to demonstrate the ability of a humanized anti-CEA antibody conjugated to a NIR fluorophore to target the primary tumor and even micro-metastases.

While cell line based models can demonstrate *in-vivo* antibody fluorescent labeling, the fluorescent signal delivered to the tumor will likely be higher than the clinical setting due to the homogeneous nature of cell lines and clonal expression of the target antigen. Subcutaneous models of patient derived tissue retain the heterogeneity of the tumor microenvironment, but they rarely induce similar systemic symptoms or metastasize [13–15]. In contrast, the pancreatic cancer PDOX model induced cachexia, ascites and jaundice, and metastasized to the liver, lung, peritoneum, and blood vessels in mice (Figure 1). The tumor retained important characteristics of the donor tumor at the microscopic level including formation glandular architecture and mucin production (Figure 2 and Supplementary Figure 1). Further detailed necropsy and histology examination showed that this tumor replicates the dissemination pattern of metastatic pancreatic cancer, with formation of satellite lesions and invasion into adjacent structures such as the spleen and abdominal wall (Figure 3). The pancreatic cancer PDOX model is clinically-relevant model to examine fluorescent antibody labeling for FGS.

After confirming that the tumor continued to express CEA seen with both western blotting and immunohistochemistry (Figure 4), we used this model to examine the tumor-targeting efficacy of the humanized anti-CEA hM5A antibody conjugated to the IRDye800CW NIR fluorophore. Previous work by our group with chimeric anti-CEA antibodies and visible wavelength dyes have shown the utility of the antibody-fluorophore conjugates with improvements in background as the fluorophores approached the NIR range [17]. The hM5A-IR800 construct is unique on a number of levels. First, the humanization prevents the formation of human-anti-chimera antibodies due to the residual murine motifs remaining on the probe. In clinical studies using chimeric anti-CEA antibodies for radioimmunotherapy imaging studies, nearly 30% of patients developed human-anti-chimera antibodies (NCT02293954, NCT00645060). Current M5A-PET imaging studies have not detected any human-anti-human antibody immunologic responses thus far (unpublished data courtesy of Dr Yazaki). Second, the parental hM5A antibody has been shown to be safe in patients, showing specificity for the CEA antigen in both pancreatic and colon cancers in the studies above. Third, the combination with IRDye800CW, a NIR fluorophore improves tissue penetration compared to visible wavelength fluorophores. This is a clinically-relevant fluorophore, as a number of FDA approved NIR imaging devices designed for indocyanine green at 800 nm can also image IRDye800CW, due to spectral overlap [18]. Phase I/II clinical trials combining cetuximab with IRDye800CW have not reported any serious adverse effects with the addition of the fluorophore [19, 20].

*In-vivo* fluorescence imaging of the pancreatic cancer PDOX using hM5A-IRDye800CW showed that the antibody-fluorophore conjugate was able to selectively target and label not only the primary tumor, but also highlighted small intra-abdominal metastases that could otherwise have been missed with only bright light imaging (Figure 5). The fluorescence intensity and contrast, as indicated by the tumor-to-background-ratio was adequate for delineating the cancerous lesions (Figure 6). However, when compared to previous work performed using the same hM5A-IR800 and a CEA-positive human pancreatic cancer cell line, BxPC3, the pancreatic cancer PDOX model showed 8-fold lower overall fluorescence intensity values and 4-fold lower contrast at 48 hours [21]. This could be due to the heterogeneity of antigen expression in a PDOX tumor compared to a uniform antigen expression from a more homogenous cell line population. This could also be due to the high serum CEA levels with shed antigen binding to a majority of the antibody-fluorophore conjugate in the serum, leading to a decreased amount of the overall probe able to reach the tumor. This possibility is supported by the two-fold increased fluorescence values at the skin in the pancreatic cancer PDOX mice compared to BxPC3 tumor-bearing mice imaged using hM5A-IR800 at 48 hours. The threshold limit of serum CEA that may affect an anti-CEA antibody reaching the tumor surface antigen is unclear. This issue that could possibly be addressed in future work by studying the amount of serum CEA shed in mouse models using pancreatic cancer cell lines as compared to PDOX models. Another possibility is to treat with a “priming” dose of unconjugated antibody to neutralize the serum antigen, followed up the fluorescently-labeled construct.

The metastases in the abdominal wall had similar fluorescence intensity compared to the tumor. The splenic lesions appear to have lower fluorescence intensity on the heat map. However these metastases were too small to reliably quantify fluorescence intensity using the CRI Maestro software. Metastases such as these can be examined under 800 nm fluorescence microscopy in future experiments. The reasons for fluorescence intensity differences are likely multifactorial: tissue permeability and perfusion issues likely play a major role. The probe is initially delivered to any tissue with perfusion and it is possible that the metastatic lesions have not yet developed the pronounced vascular supply of the primary tumor. This could lead to restricted probe delivery, decreased antibody-antigen binding and decreased fluorescence at the metastatic lesions. It could be attributed to antigenic shift leading to differential antigenic density in the metastatic lesions since metastases are further de-differentiated It is likely not attributable to tissue depth as the splenic metastases were surface lesions.

Fluorescence targeting using hM5A-IR800 produces a non-specific signal in the liver and the bladder which has been seen in our previous work [21]. Our initial study with this probe shows that it washes out to negligible levels in 48 hours, but it is not an ideal one for detection of liver metastases. Other antibody-fluorophores with limited liver accumulation would be preferable and those are actively under evaluation in our lab at this time.

Despite these issues, the images obtained using hM5A-IR800 as compared to bright-light images in the pancreatic cancer PDOX model highlight the potential of fluorescently-labeled antibodies to visualize pancreatic cancer. Pancreatic cancer is a malignancy with a dense fibrotic stroma which may necessitate a high fluorogenic potential to deliver a targeted signal. Here, the probe was able to highlight the tumor and its metastatic lesions. While the work showed proof-of-concept feasibility, it was not designed to test sensitivity and specificity of the probe. The pancreatic cancer PDOX model is an appropriate platform for further experiments such as sensitivity and specificity testing of antibody-fluorophore conjugates for FGS.

Fluorescent hM5A-IR800 has clinical relevance to determine resection boundaries of pancreatic cancer for FGS, and detect peritoneal disease during diagnostic laparoscopy where radiographically-occult metastases can be encountered up to 10% of the time despite the use of high resolution computed tomography scans [22]. The use of this technology could decrease the rates of positive pancreatic margins and increase the rates of detection of peritoneal disease. Since these targeted fluorophores give information not only about the localization of the tumor, but also its antigenic expression, they may indicate shifts in antigenic expression after neoadjuvant therapies, Further studies will need to be performed to examine the changes of fluorescence signal in this setting.

Humanized anti-CEA antibody conjugated to an NIR-fluorophore is a clinically-promising conjugate for imaging pancreatic cancers, both at the primary location as well as metastases. The hM5A-IR800 probe selectively targeted and labeled cancerous lesions in a clinically-relevant metastatic pancreatic cancer PDOX model. It was able to delineate lesions that could otherwise have been missed with only bright light visualization.

## MATERIALS AND METHODS

### Animal care

Immunocompromised nude nu/nu mice were maintained in a barrier facility on high-efficiency particulate air (HEPA)-filtered racks at AntiCancer Inc. Mice were maintained ad lib on an autoclaved laboratory rodent diet (Teckland LM-485; Western Research Products, Orange, CA, USA) and kept on a 12 hour light/ 12 hour dark cycle. All surgical procedures and intravital imaging were performed with the animals anesthetized by intramuscular injection of an anesthetic cocktail composed of ketamine 100 mg/kg (MWI Animal Health, Boise, ID, USA), xylazine 10 mg/kg (VWR, Brisbane, CA, USA), and acepromazine 3 mg/kg (Sigma, Saint Louis, MO, USA). Tumor bearing mice were monitored twice a week. All animal studies were conducted in accordance with the principles and procedures outlined in the NIH Guide for the Care and Use of Animals under PHS Assurance Number A3873-1.

### Establishment of a patient derived orthotopic xenograft mouse model

Samples from pancreatic cancers of patients undergoing surgical resection at the University of California San Diego (UCSD) Medical Center under an Internal Review Board (IRB) approved protocol #090401. Patients were consented for tissue collection and research by the UCSD Biorepository and Tissue Technology Program at their clinic visit prior to surgery. Tumor fragments were collected and implanted subcutaneously over the flanks of nude mice. Subcutaneous tumors were monitored twice a week and allowed to grow for 4–8 weeks to develop patient derived xenograft mouse models (PDX). Once the subcutaneous tumors were large enough to supply adequate tumor for orthotopic implantation, approximately 7–10 mm, the subcutaneous tumors were harvested and surgically engrafted onto the pancreatic tail of recipient nude mice using a surgical orthotopic implantation (SOI) technique developed for pancreatic cancer [16].

The pancreatic cancer PDOX was established from a specimen obtained during a liver biopsy of a patient with metastatic pancreatic cancer and it was selected for further characterization. Pre-operative serum CEA levels in the patient were greater than 300 ng/mL. In the establishment and observation group (*n* = 10), orthotopic tumor bearing mice were monitored twice weekly until development of ascites (*n* = 2) and/or jaundice (*n* = 1) as evidenced by skin color changes. These mice were then monitored daily for signs of pain/distress, impaired mobility, cachexia, and sacrificed for clinical indications of morbidity. Upon sacrifice, tissue from these mice were collected for histology.

### Antibody conjugation

The humanized hT84.66-M5A (hM5A) mAb was established by grafting the CDR region of the murine mT84.66 mAb onto a human anti-p185HER2 antibody (Trastuzumab) framework, as previously described [23]. Purified hM5A antibody was conjugated with NHS-IRDye800CW (LI-COR Biosciences, Lincoln, NE, USA) at 10-fold molar excess of dye at room temperature for 1 hour. Absorbance at 280 nm was used to determine concentration of the fluorophore-conjugated antibody. Final concentration of antibody-dye conjugate was 5.7 mg/mL. Mass spectrometry was used to determine an average of 6 dye molecules per IgG.

### Histology

Tumor tissue was removed along with surrounding normal tissue at the time of mouse necropsy including primary tumor and abdominal metastases. Tissue was harvested to confirm pancreatic origin of intra-abdominal metastases and to detect sub-clinical lesions. The following organs were harvested: primary tumor, pancreas, small and large bowel, liver, lung, spleen, abdominal wall, abdominal lymph nodes. The tissue was fixed in and embedded in paraffin. Tissue blocks were sectioned at 3 µm and stained with hematoxylin and eosin (H&E) per standard protocols. Immunohistochemistry was performed per standard protocols. Slides were incubated with hM5A as a primary antibody. Goat anti-human immunoglobulin horseradish peroxidase labeled antibody sc-2453 (Santa Cruz Biotech, Dallas TX, USA) was used as a secondary antibody. Horseradish peroxidase was visualized by a diaminobenzidine (DAB) chromogenic reaction. Light microscopy of slides was performed with an Olympus microscope equipped with the Olympus DP27 camera and CellSens software (Olympus Co, Scientific Solutions Group, Waltham, MA, USA). Interpretation of the histologic slides was performed by an experienced pathologist (BD).

### *In-vivo* fluorescence imaging studies

In the fluorescence imaging group (*n* = 3), mice with pancreatic cancer PDOX were injected intravenously with 75 µg of M5A-IR800. Mice were imaged with the Maestro CRI imaging system (Perkin Elmer, Waltham, MA, USA) 48 hours after injection. Images were acquired at the IRDye800CW wavelength (excitation 778 nm, emission 800 nm). Fluorescence intensity was quantified at the following locations: primary tumor, adjacent truncal skin, liver, and satellite lesions. Fluorescence intensity was adjusted for background noise by subtracting the peak fluorescence intensity at the adjacent truncal skin with the peak fluorescence intensity at the primary tumor. The tumor-to-background ratio was determined as a ratio of peak fluorescence intensity of the primary tumor compared to adjacent truncal skin. Fluorescence heat map intensity image was created using the Maestro CRI software.

## SUPPLEMENTARY MATERIALS FIGURE AND TABLE



## Abbreviations

CEA: Carcinoembryonic antigen
FGS: Fluorescence-guided surgery
hM5A: humanized anti-CEA antibody
H&E: Hematoxylin and eosin
IHC: Immunohistochemistry
NIR: Near-infrared
PDX: patient derived xenograft
PDOX: Patient-derived orthotopic xenograft
SOI: Surgical orthotopic implantation
