# Supplementary material for: Fluorescent humanized anti-CEA antibody specifically labels metastatic pancreatic cancer in a patient-derived orthotopic xenograft (PDOX) mouse model
Source: Oncotarget. 2018 Dec 18;9(99):37333–42. doi: 10.18632/oncotarget.26484 (PMC6324662; doi:10.18632/oncotarget.26484)
Supplement: Supplementary file 1 [file oncotarget-09-37333-s001.pdf]

Fluorescent humanized anti-CEA antibody specifically labels metastatic pancreatic cancer in a patient-derived orthotopic xenograft (PDOX) mouse model

SUPPLEMENTARY MATERIALS

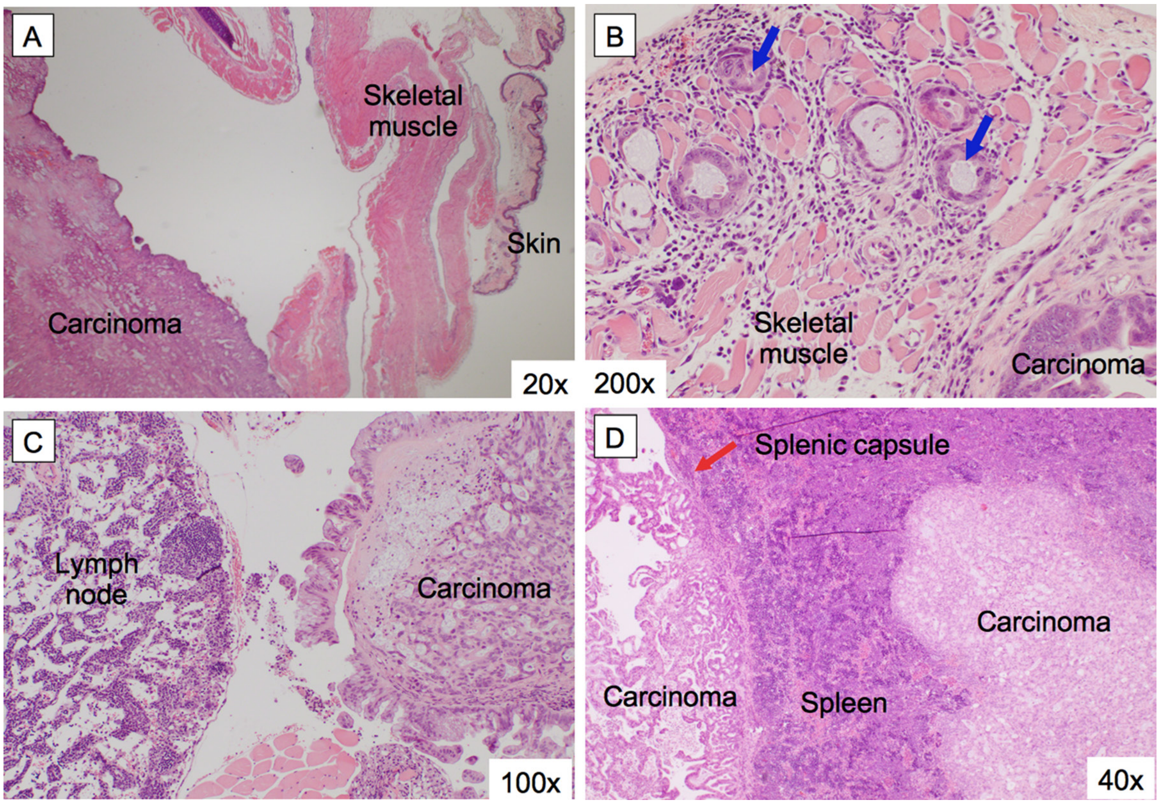

Supplementary Figure 1: Histology of the pancreatic cancer PDOX. Hematoxylin and Eosin (H&E) stain of tumors implanted into the pancreatic tail were consistent with that of the original patient's tissue specimen, a high-grade pancreatic adenocarcinoma (**A**). On higher magnification, there were areas invading into normal pancreatic acini, intermittent areas of glandular formation and mucin production, and diffuse sheets of cancer cells were prominent (**B**). There were pancreatic ducts with crowded vesicular nuclei and an area of invasion of the myoepithelial layer (**C**, black arrow). Multiple mitotic figures were present (**D**, red arrowheads).

Supplementary Table 1: Tumors and metastases developed in the established and observation cohort

Mouse	Primary tumor	Ascites	Jaundice	Liver met	Lung met	Peritoneal Met	Abd wall met	Splenic met	SB met	RP met
1	1		X	1		8			1	
2	1	X				10	1			
3	1				1*		2	6		1
4	1	X				6				
5	1						1	2		
6	1									
7	1						1*			
8	1							4		
9	1								1*	
10	1									

*Indicates microscopic lesions.

The ten mice evaluated long term had diverse tumors. 6/10 mice had grossly positive metastases on laparotomy. Two had microscopically positive deposits in the abdominal wall and small bowel (indicated by asterisks). One had a microscopically positive metastasis in the lung.
